# Towards feminist energy geographies: A critique of masculinist core-periphery binaries

**DOI:** 10.1177/20438206251364986

**Published:** 2025-08-10

**Authors:** Saska Petrova

**Affiliations:** 1 5292 University of Manchester, UK

**Keywords:** Feminist energy geographies, precarity, permeability

## Abstract

This commentary offers a brief and sympathetic critique of Ptak et al.'s (2026) argument that energy geographies is a marginal subdiscipline in human geography. While agreeing with the authors’ positioning of human geography as ‘an integrative and convergent field’, I question the notion of a centre-periphery binary within the discipline. I argue in favour of a feminist energy geographies approach by foregrounding the diverse forms of inter- and intra-disciplinary knowledge production. The commentary contends that masculinist framings of core-periphery can act as objects of power and marginalisation.

## Introduction

In June 2022, our ‘Domesticity, gender and environmentalism’ project team at the University of Manchester co-hosted a workshop on feminist approaches to energy injustices, with geographers and energy social science researchers from across the world. The workshop led to a series of inspiring discussions on the insufficient recognition of researcher positionality, alongside diverse (often trans- and/or inter-disciplinary) identities. This was found to be particularly true in relation to how our scholarly identities shape the work that we do: a statement very much in line with Massey's (2001) point that geographers often possess hybrid identities in terms of ‘counterpositional, A/not-A binaries’ over time.

Following from the workshop – and other similar events – while being inspired by feminist approaches and epistemologies in other subdisciplines in Geography and beyond, an increasing number of fellow geographers have started collaborating towards the development of feminist energy geographies. One of the key motives has been the need to unsettle the dominancy of masculinist binary framings of concepts used in Geography and energy geographies research, such as ‘home/domestic vs. public’ (Larrington-Spencer et al., 2021), ‘centre vs. core’ (Domosh, 1998; Kaika, 2004), ‘Global South vs. Global North’ (Jehlička and Jacobsson, 2021), ‘female/women vs. male/men’ (Phillips and Petrova, 2021). These binaries are, of course, more general and have been criticised by feminist scholars in Geography and related fields (Blunt and Dowling, 2006; Staeheli et al., 2013). Historically, one of the key problems with binarism in Geography is that concepts of ‘core’ and ‘periphery’ – and associated claims for continuing or revived divisions – cannot capture the inter- and intra-disciplinary intellectual richness of geographical research (Agnew, 1988).

## Energy geographies – a vibrant, interstitial space

In contrast to the growing body of feminist energy geography approaches, Ptak et al. (2026) can be read as an attempt of intra-disciplinary (re)ordering that resets processes of disciplinary ‘peripheralisation’ (Kühn, 2015) by (sub)disciplinary (re)positioning, and the (re)introduction of hierarchical and polarised disciplinary boundaries. Even if we entertain the core-periphery intellectual organisation of Geography for a moment, it would be difficult to discuss the ‘centrality’ of energy geographies based on a term borrowed from economic geography (Pugh and Dubois, 2021). I agree with Ptak et al. (2026) that energy geographies are an integrative and inclusive academic subdiscipline built on the scholarly foundations of diverse theories, approaches, and methodologies. Nevertheless, subdisciplines in Geography do not exist in isolation from each other. The strength of energy geographies lies in their relations and complementarity with other subdisciplines within and outside the bounds of Geography.

Recent years have seen multiple instances of successful cross-(sub)disciplinary collaborations within and beyond the field – an illustrative case is provided by the Royal Geographical Society's Energy Geographies Research Group webinar series, combining energy geographies with feminist, decolonial, and digital geographies. Such interactions have been evident at other key geographical conferences and events: Ptak et al. (2026) themselves mention that Petrova (2014) recorded over 100 papers across 25 sessions at the American Association of Geographers Annual Meeting in 2014, demonstrating the growing importance of energy geographies. Nevertheless, the authors find it ‘critical to note that while supported largely by the Energy and Environment Specialty Group, many papers were presented as a part of thematically broader sessions in collaboration with (or hosted by) other specialty groups’ (Ptak et al., 2026). This is precisely why I would argue that Geography is a collaborative discipline, and such joint efforts create fertile platforms for messy, but often exceptionally innovative (co)productions of knowledge.

Ptak et al.'s (2026) core-periphery argument is potentially also incompatible with another point that the authors make later in the paper. Namely, they identify three primary reasons for the continued marginalisation of energy geographies, including: (a) the colonial-capitalist politics of knowledge and production relations; (b) definitional ambiguity and discursive disarmament; and (c) disciplinary silos and selective categorisation (Ptak et al., 2026). While I would agree that those reasons are strongly valid when it comes to the politics of knowledge production in Geography and adjacent terrains, the third contingency identified by the authors is exactly what geographers have tried to avoid by supporting trans-disciplinary research and collaborating with colleagues from other (sub) disciplines.

Ptak et al. (2026) ask, ‘why after the raft of scholarship and trajectory of energy geographies during the aforementioned boom period has progression stalled?’. It would have been useful to see evidence that would demonstrate either the ‘boom’ or the ‘stalled’ progression of energy geographies scholarship. By attributing the subdiscipline's peripheral position to its internal shortcomings – without critically interrogating the broader structural and epistemological hierarchies that produce such marginalisations (if they exist) – the authors implicitly uphold the binary centre-periphery model, otherwise widely critiqued not only by feminist geographers but also by scholars in economic (Pugh and Dubois, 2021), political (Amoore, 2020), and critical geography more broadly (Castree, 2022).

## The future of energy geographies is feminist

In feminist geography, the centre-periphery dualism is often considered masculinist because it reflects and reinforces hierarchical, binary, and patriarchal ways of thinking that mirror masculine/feminine dichotomies (Massey, 1994). Centre-periphery epistemologies and positionings undermine diverse ways of knowing and producing knowledge (Rose, 1993). Peripheral spaces are often feminised and framed as sites of emotional, reproductive labour and informal work, while ‘centres’ are masculinised as spaces of formal, rational and productive work (McDowell, 1997). Feminist thinking casts spatiality – and the positioning of (sub) disciplinary knowledge – as multidimensional and hybrid.

In their intervention, Ptak et al. (2026) still suggest three potential pathways that could advance and ‘centre’ energy geographies. While their desire to develop the subdiscipline is commendable, I would question the call for ‘centring’ research in relation to an imagined ‘canonical’ heartland of Geography. Regardless of how much scholars try to remain open-minded and careful with citations and referencing, the process of labelling some forms of knowledge as ‘canonical’ can generate an exclusionary dynamic that establishes, to use Ptak et al.'s (2026) terminology, ‘centres’ of power vs. ‘peripheries’ of marginal scholarship. The ‘centre-periphery’ positioning reifies ‘the core’ and establishes it as an object of power in and of itself.

The feminist notions of (energy) ‘precarity’ and ‘permeability’ offer potential ways of transcending core-periphery dichotomies. Conceptually, ‘precarity’ has been used to depict the politically-induced vulnerability and marginalisation of people and communities, while highlighting their agency in Geography more broadly (Strauss, 2018; Vasudevan, 2014; Waite, 2009), and in energy geographies more specifically (Phillips and Petrova, 2021; Stock and Sareen, 2024). ‘Permeability’ has been used to reflect on how geographical formations are constantly reshaped and redefined through multi-faceted knowledges, experiences, legacies, and narratives (Larrington-Spencer et al., 2021; Massey, 2005). Additionally, feminist work on the ethics of care and caring-with others, including more-than-human others (Fredriksen, 2021; Petrova, 2023), can help shift dominant knowledge systems. For example, feminist decolonial scholarship (Noxolo and Hamis, 2023; Sultana, 2022) calls for re-examining power relations and imbalances inherent in the process of constructing knowledge. This body of work helps unsettle canonical epistemologies that are produced by centres of power (Hoicka, 2023).

To conclude, Ptak et al. (2026) have made a useful intervention and provocation to get us thinking about the role of energy geographies in the broader Geography landscape. However, I have questioned some of the underlying premises that their argumentation is based on – particularly the ‘core-periphery’ positioning. I have contended that we should think outside the confines of ‘canonical’ scholarship – and its problematic binaries – given the hybrid and multidimensional nature of knowledge production in Geography and beyond. Thanks to their inherent permeability and precarity, (feminist) energy geographies offer a progressive space in which identity and spatial relationships can be seen as fluid, interrelated, and non-hierarchical.
